# Regulating Gut Microbiome: Therapeutic Strategy for Rheumatoid Arthritis During Pregnancy and Lactation

**DOI:** 10.3389/fphar.2020.594042

**Published:** 2020-11-11

**Authors:** Yao Yao, Xiaoyu Cai, Weidong Fei, Fujia Ren, Fengmei Wang, Xiaofei Luan, Fengying Chen, Caihong Zheng

**Affiliations:** ^1^ Department of Pharmacy, Women’s Hospital, School of Medicine, Zhejiang University, Hangzhou, China; ^2^ Department of Pharmacy, Hangzhou First People’s Hospital, Hangzhou, China; ^3^ Department of Pharmacy, Hangzhou Women’s Hospital, Hangzhou, China

**Keywords:** gut microbiome, rheumatoid arthritis, pregnancy, lactation, treatment

## Abstract

Rheumatoid arthritis (RA) is an autoimmune disease characterized by synovial inflammation and bone destruction. Microbial infection is considered to be the most important inducement of RA. The pregnancy planning of women in childbearing age is seriously affected by the disease activity of RA. Gut microbiome, related to immunity and inflammatory response of the host. At present, emerging evidence suggested there are significant differences in the diversity and abundance of gut microbiome during pregnancy and lactation, which may be associated with the fluctuation of RA disease activity. Based on these research foundations, we pioneer the idea of regulating gut microbiome for the treatment of RA during pregnancy and lactation. In this review, we mainly introduce the potential treatment strategies for controlling the disease activity of RA based on gut microbiome during pregnancy and lactation. Besides, we also briefly generalize the effects of conventional anti-rheumatic drugs on gut microbiome, the effects of metabolic changes during pregnancy on gut microbiome, alteration of gut microbiome during pregnancy and lactation, and the effects of anti-rheumatic drugs commonly used during pregnancy and lactation on gut microbiome. These will provide a clear knowledge framework for researchers in immune-related diseases during pregnancy. Regulating gut microbiome may be a potential and effective treatment to control the disease activity of RA during pregnancy and lactation.

## Introduction

Rheumatoid arthritis (RA) is an inflammatory autoimmune disease characterized by synovitis and bone destruction (Cai et al., 2020). The pathogenesis of RA is not clear, but it is generally considered to be related to the interactions between genetic and environmental factors (Scherer et al., 2020). And at present, microbial infection is considered to be the most important inducement of RA (Li et al., 2013). RA is prevalent among women, the ratio of males to females is about 1:3, and it mostly occurs between 20 and 55 years old (Croia et al., 2019). In the past decades, the exploration between RA and women indicated that the disease activity of RA fluctuate apparently during pregnancy and lactation. The disease activity of some patients with RA decreased during pregnancy, and some increased during lactation (de Man et al., 2008; Jethwa et al., 2019; Chen et al., 2020). These conditions seriously affect family planning and the health management of women in childbearing age.

Pregnancy is a complicated and delicate process, in which the maternal hormone levels, immunity and metabolism change conspicuously to ensure the normal growth and development of the fetus (Mu et al., 2019). As early as 1931, scholars began to study the relationship between RA disease activity and pregnancy or lactation. Here, a retrospective analysis based on objective markers of RA disease activity and related scores in 2019 showed that the disease activity of 60.3% of pregnant women with RA is improved during pregnancy and that of 46.7% is increased during lactation (Jethwa et al., 2019). What causes this phenomenon? Currently, it is generally believed that this is mainly associated with hormone levels of mother. Briefly, the level of estrogen with the anti-inflammatory effect increased significantly during pregnancy and decreased rapidly during lactation (Ince-Askan et al., 2017), which might be one of the important factors that cause the fluctuation in RA disease activity during pregnancy and lactation (de Jong and Dolhain, 2017). But this cannot explain the fact that many women are still having active disease activity during pregnancy and the proportion of improvement during pregnancy has gradually decreased for decades. The disease activity of RA during pregnancy and lactation severely affects the outcome of mother and fetus and postpartum health management (Bermas, 2017). However, apart from hormonal effects, the current understanding of the relationship between pregnancy or lactation and RA is very limited. Therefore, a clear demonstration of the impact of pregnancy and lactation on RA disease activity and its mechanisms is particularly important for managing the health of pregnant women with RA.

In recent years, the role of gut microbiome in the host health during pregnancy and lactation has received widespread attention. The maternal gut microbiome undergoes tremendous changes throughout pregnancy and lactation (Gomez-Arango et al., 2016b; Mutic et al., 2017; Stanislawski et al., 2017). This provides a novel idea for exploring the relationship between pregnancy or lactation and RA disease activity. During pregnancy and lactation, there are significant changes in the diversity, species richness, and composition of the maternal gut microbiome (Nuriel-Ohayon et al., 2016; Mu et al., 2019). The human gut microbiome is complex and composed of more than 500–1,500 different bacteria, archaea, fungi and viruses (de Brito Alves et al., 2019; DeMartino and Cockburn, 2020; Weersma et al., 2020). They are not only participating in the digestion and absorption of food but also play an important role in regulating immunity and metabolism of the host (Guerreiro et al., 2018; Peterson et al., 2020; Singh, 2020). Gut microbiome disorder leads to changes in microbial metabolites and increased permeability of the intestinal mucosa. These usually disrupt the balance between non-self antigen tolerance and immunity, which may promote antigen absorption and increase the persistence or worsening of immune-mediated diseases like RA (Morrison and Preston, 2016; Guerreiro et al., 2018; Picchianti-Diamanti et al., 2018).

More and more evidence showed that gut microbiome is involved in the formation of adaptive immunity and autoimmunity (Van de Wiele et al., 2016; Agorastos and Bozikas, 2019). The balance between helper T cell subsets and regulatory T cells is to be influenced by specific gut microbiome (Wu et al., 2010). For example, the colonization of *segmental filamentous bacteria* in mice can induce the production of Th17 cells. In contrast, *Clostridia* can induce the production of regulatory T cells, thereby suppressing the autoimmune response (Lee and Kim, 2017). Additionally, gut microbiome can directly or indirectly bind NOD-like receptors (Nod-like receptors) and/or Toll-like receptors (TLRs) to activate the immune system and produce metabolites of short-chain fatty acids (Short-chain fatty acids) (Hasegawa et al., 2006; Reichardt et al., 2014). Moreover, based on the study of gut microbiome at present, probiotics and specific gut microbiome metabolites are applicable in the treatment of various autoimmune diseases (Weis, 2018; Marietta et al., 2019).

In this review, we outline the role of gut microbiome in RA, specific changes in gut microbiome during pregnancy or lactation in recent years and the possibility of improving RA disease activity during pregnancy or lactation by regulating gut microbiome. Besides, we also briefly generalize the impacts of conventional anti-rheumatic drugs on gut microbiome, the effects of metabolic changes during pregnancy on gut microbiome, the outcome of mothers and fetuses in pregnant women with RA (Preterm delivery, Cesarean section, small for gestational age), and the anti-rheumatic drugs commonly used during pregnancy and lactation. This review will provide a clear knowledge framework for researchers in the field of pregnancy-related immune diseases. Regulating gut microbiome might be a potential and effective treatment to control the disease activity of RA during pregnancy and lactation.

## The Role of Gut Microbiome in Rheumatoid Arthritis

### Gut Microbiome in the Etiology of Rheumatoid Arthritis

RA is an inflammatory autoimmune disease characterized by synovial inflammation and bone destruction (Yao et al., 2018; Yao et al., 2019). The pathogenesis of RA is complicated, including the interaction between innate immune response and acquired immune response, which involves the formation of self-reactive T cells, antigen presentation and the formation of autoantibodies. These autoantibodies may come from various parts of the body, such as the gastrointestinal tract, and eventually enter the blood circulation, causing systemic joint inflammation (Malmstrom et al., 2017; Favalli et al., 2019).

**FIGURE 1:**
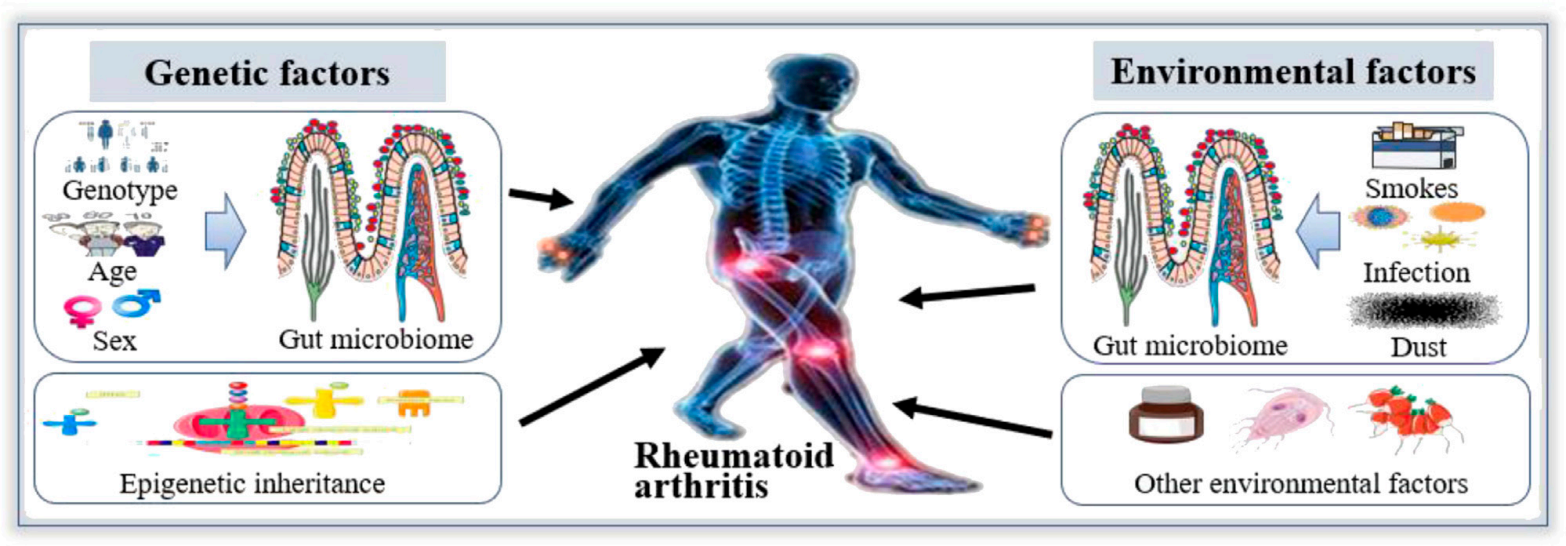
Gut microbiome in the etiology of Rheumatoid arthritis (RA). The etiology of RA is mainly related to environmental factors and genetic factors. Among the environmental factors, smoking, infection and dust can affect the structure of gut microbiome to participate in the occurrence and development of RA. Among genetic factors, the composition of gut microbiome is associated with genotype and sex, which is linked to the occurrence and development of RA. Therefore, gut microbiome play an important role in the etiology of RA.

The studies showed that the development of RA is based on heredity and epigenetics, but environmental factors also play an important role, such as cigarette smoke and dust contact, especially the microbiome representing the “internal” environment (Figure 1) (Amariuta et al., 2020; Scherer et al., 2020). And the susceptibility of RA is related to environmental factors, such as smoking and infection, which may be mediated by gut microbiome (Klareskog et al., 2006; Luckey et al., 2013). In particular, genotype and sex are strong determinants of gut microbiome, although gut microbiome changes dynamically with age (Gomez et al., 2012; Morgan et al., 2012). In humans, age-related alterations of gut microbial composition are associated with enrichment of pathobionts (Yatsunenko et al., 2012; Rampelli et al., 2013). These changes may cause an increase or loss of intestinal metabolic function, leading to disorders of gut microbiome. Additionally, smoking has been shown to affect the diversity of gut microbiome and quitting smoking will increase the diversity of gut microbiome (Biedermann et al., 2013; Savin et al., 2018). These findings indicate that the decrease in the diversity of gut microbiome is one of the reasons for the development of RA.

With the increased understanding of gut microbiome, there has been a wide area of related researches, especially in autoimmune diseases. The evidence supporting the association of gut microbiome with RA is as follows: 1) Compared with the control group, the composition of gut microbiome changed in early RA patients, the abundance of certain bacteria in the genus Bifidobacterium and *Bacteroides* decreased, and genus Prevotella increased significantly (Vaahtovuo et al., 2008; Bernard, 2014; Jeong et al., 2019). 2) In the germ-free model, parenteral injection of cell wall fragments of exogenous intestinal bacteria can induce arthritis (Scher et al., 2013; Bernard, 2014). 3) Partial gut microbiome of clinical RA patients restored to normality after antirheumatic treatment (Zhang et al., 2015; Bodkhe et al., 2019). 4) Existing reports have shown that diet could affect the activity of inflammation, and high-fat diet has a negative effect on intestinal permeability (Skoldstam et al., 2003; Rohr et al., 2020). 5) Many drugs for the treatment of RA have antibacterial properties, such as minocycline, chloroquine, roxithromycin and sulfadiazine (Ogrendik, 2009; Ogrendik and Karagoz, 2011). 6) The alpha diversity index of gut microbiome in the feces of RA patients is significantly lower than that of healthy people. The bacterial genera *Bacteroides* and Escherichia-Shigella were more abundant in RA patients. In contrast, *Lactobacillus*, *Odoribacter*, *Enterobacter*, and *Alloprevotella* were less abundant in RA patients than that in healthy people (Drago, 2019; Sun et al., 2019). Based on the above facts, we conclude that gut microbiome may be closely related to RA, and the imbalance of gut microbiome may be one of the causes of RA.

### The Mechanism of Gut Microbiome in Rheumatoid Arthritis

The studies of gut microbiome in animal models, the effects of diet and probiotics on the extent of inflammatory activity support the potential role of gut microbiome in the pathogenesis of RA (Badsha, 2018; Jubair et al., 2018). Researches in RA patients or animal models indicated that the abundance of many genera of gut microbiome increased or decreased significantly compared with the control group (healthy people or animal). The influence mechanism of gut microbiome on the pathophysiology of RA may be multifactorial, and it mainly includes the following aspects: the ability to produce citrullinization of peptides by enzymatic action, activation of antigen-presenting cells through an effect on TLRs or NLRs, antigenic mimicry, control of host immune system (triggering T cell differentiation), alterations in permeability of intestinal mucosal, and increase of T helper type 17-mediated mucosal inflammation [Table 1 (Round et al., 2011; Marietta et al., 2016; Teng et al., 2016; Pahari et al., 2017; Guo et al., 2019; Li et al., 2020; McHugh, 2020; Tajik et al., 2020)].

**TABLE 1:** The mechanism of gut microbiome in RA (animal experiment).

Experimental model	Bacteria involved	Analytical method	Mechanism	References
K/BxN mice, induced arthritis	*Segmented filamentous bacteria (SFB)*	*SFB*-specific 16S rRNA quantitative PCR	SFB enhance autoimmune arthritis, reflected by elevated auto-ab, GC, and tfh cell responses. SFB induce Peyer’s patch T follicular helper cell differentiation by limiting the access of IL-2 to Peyer’s patch CD4^+^ T cells	(Teng et al., 2016)
Wistar female rat, collagen-induced arthritis	Rarefaction and α diversity of gut microbiome	16S rRNA gene sequencing	Saponins alleviation collagen-induced arthritis in rats by regulating metabolites of gut microbiomes (short-chain fatty acids, C_2_–C_6_)	(Guo et al., 2019)
Experimental arthritis	Particular bacteria genera	Not applicable	Microbiota-derived metabolites help regulatory B cells suppress arthritis	(McHugh, 2020)
Female RA patients and collagen-induced arthritis mice	Gut microbiota composition	16S rRNA microbiome analysis	Breach in intestinal permeability is mediated by the microbiota. Reducing intestinal barrier permeability attenuates rheumatoid arthritis	(Tajik et al., 2020)
SD rat, collagen-induced arthritis	*Bacteroides* and *Bacillus, α* diversity of gut microbiome	16S rRNA gene sequencing	Gut microbiota regulated the aryl hydrocarbon receptor pathway through indole metabolism. Regulating host immunity	(Li et al., 2020)
Expert protein analysis system	The microbial peptides	Immunoinformatics tools	Molecular mimicry, antigenic mimicry	(Pahari et al., 2017)
Transgenic mice, collagen-induced arthritis	*Prevotella histicola*	Isolation of *Prevotella species*, and administered enterally	Enteral exposure to *Prevotella histicola* suppresses arthritis via mucosal regulation	(Marietta et al., 2016)
Different TLR-deficient mice	*Bacteroides fragilis*	*Bacteroides fragilis* colonization	Toll-like receptor 2 on CD4^+^ T cells is required for *Bacteroides fragilis* colonization of a unique mucosal niche in mice during homeostasis	(Round et al., 2011)

RA, Rheumatoid arthritis. The possible mechanisms of gut microbiome in RA (only animal experiment) are showed in this table.

The evidence related to gut microbiome participating in the pathogenesis of RA all came from animal experiments based on the current research. In humans, it is only proven that there is a difference in gut microbiome between RA patients and healthy people, which cannot clarify the causal relationship between RA and gut microbiome. To predict the role of gut microbiome in the pathogenesis of RA, an improved understanding related to gut microbiome of the ecology and interactions with other microorganisms and hosts is still needed. Furthermore, the interaction between gut microbiome and the immune system might be the most likely influencing factor for gut microbiome to participate in the pathogenesis of RA, especially the influence of gut microbiome on Th17 cells. And the in-depth study of animal experiments will lay a solid foundation for clinical research.

### Rheumatoid Arthritis Treatment Based on Gut Microbiome

The pathogenesis of RA is considered to be the result of the interaction between genetics and the environment. The drugs used to treat RA are mainly disease-modifying antirheumatic drugs (DMARDs), which play an important role in controlling the symptoms and progression of the disease (Burmester and Pope, 2017). Recently, more and more evidence showed that gut microbiome regulates the host immune system directly or indirectly, for example, some gut microbiome in RA patients returned to normal after treatment with DMARDs (Bodkhe et al., 2019). Microbiome refers to a collection of genomes within an ecological community of microorganisms (Boon et al., 2014). It is difficult to clarify the role and mechanism of gut microbiome in human diseases due to only about 1% of microbes are culturable (Prakash et al., 2013). Fortunately, the development of DNA sequencing technology has made it possible to study unculturable microbes (The Human Microbiome Project Consortium, 2012; Ranjan et al., 2016). Gut microbiome has a strong metabolic potential and an important influence on the stability and activity of drugs that enter the intestine (Vande Voorde et al., 2014; Lehouritis et al., 2015). For example, the action of sulfasalazine (SSZ, a drug used to treat RA) must depend on anaerobic bacteria in the colon (Sousa et al., 2014). Moreover, drug treatment also has a significant effect on the composition of gut microbiome in the host (Sousa et al., 2008; Maier et al., 2018). Therefore, alterations of gut microbiome caused by drug affect the therapeutic effect and the host’s immune response [Table 2 (West et al., 1974; Kanerud et al., 1994; Angelakis et al., 2014; Zhang et al., 2015; Chen et al., 2016; Picchianti-Diamanti et al., 2018; Zhou et al., 2018)].

**TABLE 2:** The effect of some DMARDs on gut microbiome.

DMARDs	Pharmacological mechanism	Species studied	Effects on gut microbiome	References
Sulfasalazine	Interferes in the conversion of arachidonic acid to prostaglandins, affects leukocyte function and production of cytokines	Human	Significant increase in *bacillus* and decrease in total erobic bacteria, *Escherichia coli* and *Bacteroides*	(Kanerud et al., 1994)
		Human	Reduction in the numbers of total nonsporing anaerobes, *Enterobacteria* and opalescentnegative clostridia	(West et al., 1974)
Methotrexate	Interferes with the synthesis of pyrimidine and purines, leads to the inhibition of lymphocyte proliferation	Balb/c mice	Decrease in *Bacteroides fragilis* post-treatment	(Zhou et al., 2018)
		Human	Reduced abundance of Enterobacteriaceae	(Picchianti-Diamanti et al., 2018)
		Human	Partially restored the gut microbiota in patients	(Zhang et al., 2015; Chen et al., 2016)
Hydroxychloroquine	Interferes with antigen processing in macrophages and its presentation to MHC class II proteins	Human	Increase in microbial species richness and diversity	(Chen et al., 2016)
		Human	Hydroxychloroquine plus doxycycline treatment led to the reduction in abundance of phylum *Bacteroidetes* and *Firmicutes*	(Angelakis et al., 2014)

DMARD, disease-modifying antirheumatic drug. The drugs used to treat RA are mainly DMARDs, which plays an important role in controlling the symptoms and progression of the disease. In DMARDs, Sulfasalazine, Methotrexate and Hydroxychloroquine have an effect on gut microbiome.

The drugs currently used to treat RA mainly include traditional DMARDs, biologic DMARDs, biologics, and alternative medicines such as herbs and probiotics (Demoruelle and Deane, 2012). Traditional DMARDs such as leflunomide, methotrexate (MTX), SSZ and hydroxychloroquine are essential in the treatment of RA (Scher et al., 2013). Compared with the control group, the gut microbiome diversity of RA patients increased and the disease activity of RA decreased after receiving these drugs (Zhang et al., 2015; Chen et al., 2016). Table 2 summarizes the effects of some DMARDs on gut microbiome (West et al., 1974; Kanerud et al., 1994; Angelakis et al., 2014; Zhang et al., 2015; Chen et al., 2016; Picchianti-Diamanti et al., 2018; Zhou et al., 2018). In general, biologic DMARDs (bDMARDs) will be considered when RA patients are not effective in the treatment of traditional DMARDs. As with traditional DMARDs, treatment with bDMARDs also causes changes in the abundance of gut microbiome (Picchianti-Diamanti et al., 2018). For example, with the treatment of etanercept (belongs to bDMARDs), the abundance of *Cyanobacteria* in the intestine increased significantly compared with untreated patients (Bodkhe et al., 2019). Chinese herbal medicine has been used to treat various incurable diseases since ancient times (Rigau-Perez, 2016). In recent years, a relationship between the therapeutic effect of Chinese herbal medicine and gut microbiome has been proved by researchers. Xiao M et al. found that the diversity of gut microbiome increased in collagen-induced arthritis mice treated with *Paederia scandens* (Chinese herbal medicine) (Xiao et al., 2018). In addition, the therapeutic effect of another Chinese herbal medicine, *Tripterygium wilfordii* (Chinese herbal medicine), used to treat RA has also been shown to be related to gut microbiome (Miao et al., 2015). After receiving the treatment of *Tripterygium wilfordii*, the abundance of *Prevotella intermedia* in the intestine of RA patients was increased (Zhang et al., 2015). What’s more, the direct application of probiotics proves the importance of regulating gut microbiome to RA (Pineda Mde et al., 2011; Vaghef-Mehrabany et al., 2014). Therefore, the treatment of RA based on gut microbiome may be a potential and effective strategy.

## Gut Microbiome in Pregnancy and Lactation

### Alterations of Gut Microbiome During Pregnancy

Pregnancy is a complex and delicate process with remarkable changes in the hormone level, immunity and metabolism of the mother to ensure the successful growth and development of the fetus (Pazos et al., 2012; Fejzo et al., 2019). The immune system and some complications during pregnancy are related to gut microbiome (Nyangahu et al., 2018; de Brito Alves et al., 2019). Several studies have proved that microbial infections such as bacteria, fungi or viruses during pregnancy are important risk factors for adverse pregnancy outcomes, including eclampsia, premature rupture of membranes, recurrent miscarriage, intrauterine growth retardation and premature delivery (Lamont, 2015; Young et al., 2015). Notably, there is a conspicuous change in the diversity of gut microbiome during the process of pregnancy (Chen et al., 2017). The whole microbial community undergoes an amazing rearrangement related to the host’s physiology and immunity (Koren et al., 2012; Paysour et al., 2019). Recently, some scholars suggested that gut microbiome remodeling during pregnancy is a positive reaction of the mother and necessary for a successful pregnancy, which may contribute to the state of the immunity and metabolism (Konstantinov et al., 2013). However, more researchers believe that changes in gut microbiome during pregnancy are harmful to pregnant women and will cause related complications (Morffy Smith et al., 2019).

The physiological changes of pregnant women are accompanied by the alteration of microbes, especially gut microbiome (Koren et al., 2012; Edwards et al., 2017). The gut microbiome of first trimester is similar in many aspects to that of healthy nonpregnant male and female controls. However, by the third trimester, the structure and composition of the community resemble a disease-associated dysbiosis that differs among women (Koren et al., 2012). Generally, pregnancy is characterized by increased bacterial load and profound changes in the composition of intestinal microorganisms (Nuriel-Ohayon et al., 2016; Smid et al., 2018). During pregnancy, the changes of gut microbiome mainly include the decrease of individual richness (α diversity), the change of specific species richness and the increase of between-subject diversity (β diversity) (Koren et al., 2012; Chen et al., 2017). Table 3 summarizes alterations of gut microbiome during pregnancy (Koren et al., 2012; Gohir et al., 2015; Smid et al., 2018; Chen Y. et al., 2019; Ji et al., 2019; Liu et al., 2019).

**TABLE 3:** Alteration of gut microbiome during pregnancy.

Bacterial Taxa (↓ low, ↑ enriched)	Research object	Relevant conclusions	Analytical method	References
*Proteobacteria* ↑, *Actinobacteria* ↑, *Faecalibacterium* ↓	Normal women third trimester VS. non-pregnant women	Gut microbiomes are profoundly altered during pregnancy	16S rRNA gene sequencing	(Koren et al., 2012)
*Clostridiales* ↑, *Desulfovibrio* ↑, *Mogibacteriaceae* ↑, *Prevotella* ↑	Normal pregnant sows VS. non-pregnant sows	Stages of pregnancy influence the gut microbiota diversity and function in sows	16S rRNA gene sequencing	(Ji et al., 2019; Liu et al., 2019)
*Firmicutes* ↓, *Bacteroidetes* ↓, *Ruminococcaceae* ↓, *Akkermansia* ↑	Normal pregnant women, from 32 weeks of gestation to antepartum VS. non-pregnant women	The gut microbiota varied during the third trimester	16S rRNA gene sequencing	(Chen et al., 2019b)
*Proteobacteria* (ET2) ↑	Healthy women, early second trimester (ET2) VS. late second trimester	Maternal microbiome biodiversity changes as pregnancy progresses	16S rRNA gene sequencing	(Smid et al., 2018)
*Akkermansia* ↑*, Clostridium* ↑*, Bacteroides* ↑, *Bifidobacterium* ↑	Normal pregnant mice VS. non-pregnant mice	Pregnancy-induced changes in the female gut microbiota occur immediately at the onset of pregnancy	16S rRNA gene sequencing	(Gohir et al., 2015)

The structure of gut microbiome is connected with pregnancy. The changes of specific flora, the analytical method used and the research object are summarized in this table.

### Factors Affecting Gut Microbiome During Pregnancy

The initial changes in the mother during pregnancy are hormone levels. Progesterone and estrogen levels rise sharply, which cause many physiological effects (Di Renzo et al., 2016; Hudon Thibeault et al., 2019). On the one hand, these hormone levels are likely to affect the structure of gut microbiome because it has been proven that the composition of microorganisms is involved in responding and regulating host hormone levels, and host hormone levels affect bacterial growth (Gomez-Arango et al., 2016a). On the other hand, microorganisms can also secrete or produce hormones. Therefore, the interaction between hormones and microorganisms is bidirectional, and the two interact with each other (Neuman et al., 2015). However, there is a lack of direct evidence for the effect of gut microbiome on these hormones and the effect of estrogen or progesterone on gut microbiome. Further research is necessary. Besides, the immune system of mothers changes during pregnancy to support the successful growth of the fetus. Many studies have proven that gut microbiome participates in the regulation of the immune system, and the possible mechanism is antigen simulation or change in intestinal permeability (Rooks and Garrett, 2016; Neuman and Koren, 2017).

Apart from hormones, the relationship between metabolic changes during pregnancy and gut microbiome has received widespread attention at present. Metabolic changes during pregnancy are very similar to those in metabolic syndrome, including changes in metabolic hormone levels, weight gain, glucose intolerance, elevated fasting blood glucose levels, low-grade inflammation, and insulin resistance (Meo and Hassain, 2016; Nuriel-Ohayon et al., 2016; Zeng et al., 2017). These changes are closely connected with the composition of gut microbiome. Table 4 summarizes the effects of different metabolic changes during pregnancy on gut microbiome (Collado et al., 2008; Santacruz et al., 2010; Gohir et al., 2015; Kuang et al., 2017; Crusell et al., 2018).

**TABLE 4:** Effects of different metabolic changes during pregnancy on gut microbiome.

Bacterial Taxa (↓ low, ↑ enriched)	Analytical method	Research object information	Relevant conclusions	References
*Actinobacteria* phylum ↑, *Collinsella* genus ↑, *Rothia* genus ↑, *Desulfovibrio* genus ↑	16S rRNA gene amplicon sequencing	Third trimester, gestational diabetes mellitus (GDM)	GDM diagnosed in the third trimester of pregnancy is associated with a disrupted gut microbiota composition compared with normoglycaemic pregnant women	(Crusell et al., 2018)
*Bacteroides* ↑, *Sta*phylococcus ↑	FISH combined with flow cytometry and qPCR	Overweight pregnant women	Gut microbiota composition and weight are linked, and mothe’s weight gain is affected by microbiota	(Collado et al., 2008)
*Allobacullum* ↑, *Sarcina* ↑, *Biophila* ↑, *Coprobacillus* ↑, *Staphylococcus* ↑, *Akkermansia* ↑, *Bifidobacterium* ↑	16S rRNA gene sequencing	Pregnant 5C7BL/6 mice with high-fat diet	Shifts in the maternal gut microbiome have been implicated in metabolic adaptations to pregnancy	(Gohir et al., 2015)
*Parabacteroides distasonis* ↑, *Klebsiella variicola* ↑, *Methanobrevibacter smithii* ↓, *Alistipes spp.* ↓, *Bifdobacterium spp.* ↓, *Eubacterium spp.* ↓	Whole-metagenome shotgun sequencing	Pregnant women with gestational diabetes mellitus	The human gut microbiome can modulate metabolic health and affect insulin resistance, and it may play an important role in the etiology of gestational diabetes mellitus	(Kuang et al., 2017)
*Bifidobacterium* ↓, *Bacteroides* ↓, *Staphylococcus* ↑, Enterobacteriaceae ↑, *Escherichia coli* ↑	Quantitative real-time PCR	24 weeks of pregnancy, overweight pregnant women	Gut microbiomes composition is related to body weight, weight gain and metabolic biomarkers during pregnancy	(Santacruz et al., 2010)

Compared with normal healthy women, the abundance and diversity of gut microbiome during pregnancy are significantly different. This may be related to the mother’s hormone levels and metabolism. The effects of metabolism on gut microbiome during pregnancy are summarized in this table.

### Alterations of Gut Microbiome During Lactation

Lactation refers to the period from a mother secretes milk from her mammary glands to the stop of breastfeeding, which generally lasts for 10–12 months. The level of prolactin in postpartum mothers increased significantly, while the level of progesterone decreased rapidly (Vieira Borba and Shoenfeld, 2019). As mentioned above, changes in hormones affect the structure of gut microbiome. At present, the study of gut microbiome in lactation is less than that in pregnancy. Unlike the fluctuations observed during gestation, the bacterial composition appeared to be relatively stable over different stages of lactation (Liu et al., 2019). Liu et al. found that Christensenellaceae, Lachnospiraceae, and *Escherichia coli* are enriched during lactation in sows (Liu et al., 2019). Besides, another analysis of gut microbiome of the sow from pregnancy to weaning showed that the abundance of *Tenericutes* during lactation is significantly increased while *Bacteroidetes* and *Fibrobacteres* are decreased (Ji et al., 2019). These studies indicate that the gut microbiome during lactation is also in a state of disorder and continues the characteristics of pregnancy. The diversity of gut microbiome during lactation is similar to that in late pregnancy, and there was no significant change over time (Jost et al., 2014). However, we need more evidence to demonstrate the characteristic of gut microbiome in lactation. The difference between gut microbiome in lactation and normal women needs to be clarified to explore the specific genus of gut microbiome during lactation. This may help us to further study the role and mechanism of gut microbiome in disease states and to manage the health of lactating women.

In addition to lactation, there are also significant differences in the composition of gut microbiome in postpartum women. Operational taxonomic units richness and Shannon index decreased from late pregnancy to postpartum regardless of metabolic status (Crusell et al., 2018; Hasan et al., 2018). However, it lack of evidence to demonstrate the difference between breastfeeding and non-breastfeeding women, and further researches are needed.

## The Treatment of Rheumatoid Arthritis During Pregnancy and Lactation Based on Gut Microbiome

### Fluctuation of Disease Activity During Pregnancy and Lactation, and Feasibility of the Treatment Based on Gut Microbiome

RA is common among women, and the ratio of men to women is close to 1:3. In women with RA, it seems to be more difficult to conceive, and the disease activity affects the mother and fetal outcome (Wallenius et al., 2011; Wallenius et al., 2012; Yao et al., 2020). For example, RA patients have a longer time to pregnancy (TTP). Table 5 summarizes some studies on pregnancy outcomes of RA patients (Nelson et al., 1992; Skomsvoll et al., 1999; Reed et al., 2006; de Man et al., 2009; Lin et al., 2010; Norgaard et al., 2010; Brouwer et al., 2015b). The disease activity of some patients usually improves during pregnancy. However, a large number of RA patients (>50%) still suffer from active diseases during pregnancy. Thus, clinical treatment is necessary, especially considering the negative correlation between active diseases and pregnancy outcomes (de Jong and Dolhain, 2017). At present, during pregnancy and lactation, antirheumatic drugs are mainly used to control disease activities. The main side effect of the approved antirheumatic drugs could increase TTP, including nonsteroidal anti-inflammatory drugs (NSAIDs) and prednisone (in a dose >7.5 mg daily) (Brouwer et al., 2015a). In addition, the use of MTX may damage fertility (Provost et al., 2014). Therefore, a treat-to-target strategy aiming for low disease activity is recommended for RA patients with the plan of conceive.

**TABLE 5:** Some studies on pregnancy outcomes of RA patients.

Number of patients	Increased risk	Study type	References
4,716 female patients with inflammatory arthritis (RA, juvenile RA, ankylosing spondylitis) vs. 1,645,029 unaffected female controls	Preterm delivery (7.1 vs. 5.6%), SGA infants (9.9 vs. 8.7%)	Retrospective (birth registry)	(Skomsvoll et al., 1999)
243 female RA patients vs. 2,559 unaffected female controls	Preterm delivery (13.6 vs. 7.2%), cesarean section (34 vs. 19.5%)	Retrospective (birth registry)	(Reed et al., 2006)
1,199 female RA patients vs. 870,380 unaffected female controls	Preterm delivery (9.2 vs. 6.2%), SGA infants (5.9 vs. 3.8%)	Retrospective (birth registry)	(Norgaard et al., 2010)
144 female RA patients vs. 605 unaffected female controls	No increased risk: spontaneous abortions, stillbirths	Case–control	(Nelson et al., 1992)
1,912 female RA patients vs. 9,560 matched controls	SGA infants (17.3 vs. 14.9%), cesarean section (42 vs. 37.7%), preeclampsia (2.7 vs. 1.2%)	Retrospective (birth registry)	(Lin et al., 2010)
152 female RA patients vs. 175,498 unaffected female controls	Instrumental vaginal delivery (17 vs. 10%), cesarean sections group with DAS28-CRP ≥3.2 (22 vs. 10% in group with DAS28–CRP <3.2%), SGA infants 3.3%	Prospective	(de Man et al., 2009)
162 female RA patients vs. general Dutch population	No increased risk: miscarriage	Prospective	(Brouwer et al., 2015b)

SGA, small for gestational age; RA, Rheumatoid arthritis. The adverse pregnancy outcomes of RA patients are summarized.

RA improves during pregnancy and relapses during lactation, but the improvement effect is not as previously thought (Gerosa et al., 2016). This results in a substantial number of RA patients with moderate to high disease activity during pregnancy (Ince-Askan and Dolhain, 2015). Therefore, how to take a scientific and reasonable plan to improve the maternal and infant outcomes and improve or control the condition of these patients has become the focus of clinical research in the field of rheumatism. The causes of the disease activity in pregnancy and lactation are still unclear at present. Through the study of pregnancy and RA and reviewing the relevant reports at home and abroad in recent years, we summarized the possible mechanisms as follows: 1) After pregnancy, the increase in progesterone and estrogen causes the level of glucocorticoid to rise, and estrogen itself also has anti-inflammatory effects. 2) The conversion of hormone secretion after pregnancy, the balance of Thl/Th2 type immune response and the activity of T cells can be indirectly regulated and suppressed by higher levels of estrogen. 3) Changes in regulatory T cell subsets can reduce the immune response to inflammation, which not only makes the mother immune tolerance to the fetus but also reduces the immune response to RA. 4) The conversion of postpartum hormone secretion makes the level of hormones with anti-inflammatory effect drop rapidly so that the pro-inflammatory effect of Th1-related cytokines is dominant. 5) Prolactin influences the negative selection of autoreactive B cells, promoting their proliferation, survival, and antibody production.

However, mechanisms of the fluctuation of RA disease activity during pregnancy and lactation are mainly based on the level of hormones. These cannot explain the decrease in improvement rate of RA during pregnancy in recent years. The improvement of RA during pregnancy can be attributed to not just a single mechanism but most likely several different pathogenic mechanisms (Ince-Askan and Dolhain, 2015; Murakawa, 2016). Controlling RA disease activity during pregnancy and lactation is challenging, especially because several antirheumatic drugs are contraindicated in pregnancy (Forger and Villiger, 2016; Ngian et al., 2016). Extensive studies on gut microbiome in RA and pregnancy revealed that gut microbiome might play an important role in pregnancy with RA. Changes in the diversity of gut microbiome during pregnancy and lactation may be one of the key factors in the fluctuation of RA disease activity. Moreover, compared with anti-rheumatic drugs, regulating the gut microbiome to control the disease activity of RA may be safer for pregnant women and fetuses. Based on the recent studies of gut microbiome between RA and pregnancy or lactation, we propose a new treatment strategy: regulating gut microbiome. Here, we summarize several methods for the treatment of RA during pregnancy and lactation based on gut microbiome.

### Therapeutic Method Based on Gut Microbiome

#### Fecal Microbiota Transplantation

Fecal microbiota transplantation (FMT) therapy refers to the transfer of fecal microbial extracts from healthy donors to diseased recipients through colonoscopy, oral, nasogastric tube, nasal duodenum, or enema, to reconstruct the complete intestinal microbial community and reverse the imbalance of microecology (Vindigni and Surawicz, 2017; Wang et al., 2019). The research on FMT could be traced back to 1958. Four patients with pseudomembranous colitis survived through FMT (Eiseman et al., 1958). At present, FMT is mainly used to combat *Clostridium difficile* infectious colitis, and the cure rate can reach 90% (Vindigni and Surawicz, 2017). Additionally, FMT is also used in other diseases, such as Parkinson’s disease, obesity and metabolic syndrome, cancer, and multiple sclerosis (Choi and Cho, 2016; Chen D. et al., 2019; Zhou et al., 2019). FMT has not been used in patients with RA. However, when discussing the potential application of this method in inflammatory arthritis, some scholars suggested that the research results of FMT in inflammatory bowel disease are related to inflammatory arthritis. Especially compared with ulcerative colitis, there is a stronger association between spinal arthritis and Crohn’s disease (Abdollahi-Roodsaz et al., 2016). Therefore, FMT may be a therapeutic method for improving RA disease activity during pregnancy after the mechanism of gut microbiome in pregnancy with RA has been elucidated.

#### Prebiotics or Probiotics Supplementation

Prebiotics is an indigestible food ingredient that selectively stimulates the growth and activity of probiotics in the intestine, thereby improving host health (Naseer et al., 2020; Ricke et al., 2020). It is currently defined as “a substrate that is selectively utilized by host microorganisms endowed with health benefits,” and its “selectivity” mainly refers to *Lactobacillus* and *Bifidobacterium* (da Silva et al., 2020). Oral prebiotics supplements, especially those added with plant polysaccharides, are effective to improve gut microbiome (Roberfroid et al., 2010). Probiotics are beneficial to human health, such as *Bifidobacterium*, *Lactobacillus* and *Yeast* (Morovic and Budinoff, 2020). Probiotics/prebiotics is widely used in the medical field to prevent or treat some diseases, and their therapeutic effect on RA has been confirmed in animals and humans (Mohammed et al., 2017). Probiotics/prebiotics intervention may be a safe and effective method to regulate gut microbiome.

#### Reasonable Use of Antibiotics

The treatment of gut microbiome also includes the use and management of antibiotics (Iizumi et al., 2017). Different kinds of antibiotics have different effects on gut microbiome (Lange et al., 2016). A study showed that the delayed onset of RA flares after the use of specific antibiotics might be mediated through the gut microbiome (Nagra et al., 2019). However, the use of antibiotics during pregnancy could affect the health of infant and the exposure of the breastfed infant to antibiotics is related to bacterial resistance and the development of gut microbiome (van Wattum et al., 2019; Zimmermann and Curtis, 2020). Therefore, the use of antibiotics to regulate gut microbiome during pregnancy should be carefully considered. And reasonable use of antibiotics can effectively control the disease activity of RA during pregnancy or lactation.

#### Gut Pharmacomicrobiomics

The ability of gut microbiome to metabolize drugs is comparable to that of the liver (Enright et al., 2016). Gut pharmacomicrobiomics reflects the influence of changes in gut microbiome on drug pharmacokinetics and pharmacodynamics (Saad et al., 2012; Doestzada et al., 2018). Most drugs have little contact with gut microbiome because they are rapidly and completely absorbed by the upper gastrointestinal tract (Kim, 2015). However, some drugs are converted into active, inactive or toxic metabolites by gut microbiome located in the ileum and colon (Wilson and Nicholson, 2017). For example, the antirheumatic drug sulfasalazine is broken down into sulfapyridine and 5-aminosalicylic acid by the bacterial azoreductase in the colon, which is not metabolized when antibiotics are given (Peppercorn and Goldman, 1972). Pharmacomicrobiomics based on gut microbiome may be a potential treatment for RA during pregnancy or lactation. Current research needs to further clarify the mechanism of interaction between gut microbiome and drugs. The intervention of enzymes produced by specific bacteria may be of great significance for the treatment of RA during pregnancy or lactation.

#### Diet Regulation

Gut microbiome could be involved in energy balance by affecting the efficiency of energy harvesting from the diet and the expression of host genes related to the regulation of energy storage and expenditure (Guo et al., 2018). It is well known that diet is closely related to obesity and hyperlipidemia. Obesity and hyperlipidemia have a great influence on gut microbiome during pregnancy. The abundance of the phylum Firmicutes was lower and *Bacteroidetes* was higher in strict vegetarians, with the genus Prevotella being increased among other changes (Franco-de-Moraes et al., 2017). A randomized controlled trial showed that differences exited in the relative abundance of several genera in pregnancy women on a vegetarian diet, specifically a reduction in *Collinsella*, *Holdemania*, and increases in the relative abundances of *Roseburia* and Lachnospiraceae (Barrett et al., 2018). Therefore, diet control may be an effective treatment method for RA during pregnancy or lactation.

## Discussion

Presently, there is limited knowledge about the fluctuation of RA activity during pregnancy or lactation in addition to hormonal effects. We speculate that the changes of gut microbiome during pregnancy are harmful to the disease activity of RA based on current studies. The improvement of the disease activity of RA during pregnancy may be the result of neutralization between the harmful effects of gut microbiome and the beneficial effects of estrogen and progesterone. From late pregnancy to lactation, the levels of estrogen and progesterone decreased rapidly, and the leading role of gut microbiome appeared, showing the recurrence of RA at this time. Therefore, regulation of gut microbiome may be a potential strategy for the treatment of RA during pregnancy and lactation. Combined with domestic and foreign literature reports, the treatment of RA based on gut microbiome mainly includes fecal microbiota transplantation, prebiotics or probiotics supplementation, reasonable use of antibiotics, gut pharmacomicrobiomics and diet regulation.

**FIGURE 2:**
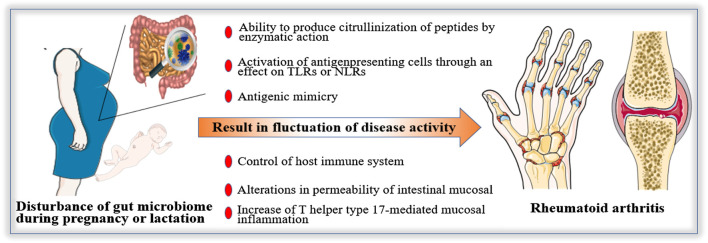
Possible mechanisms of gut microbiome in Rheumatoid arthritis (RA) during pregnancy and lactation. The fluctuation of disease activity of RA during pregnancy and lactation may be affected by gut microbiome. The possible mechanisms are as follows: 1) The ability to produce citrullinization of peptides by enzymatic action. 2) Activation of antigenpresenting cells through an effect on Toll-like receptors or Nod-like receptors. 3) Antigenic mimicry 4) Control of host immune system (triggering T cell differentiation). 5) Alterations in permeability of intestinal mucosal. 6) Increase of T helper type 17-mediated mucosal inflammation.

However, current knowledge in this field is limited. Most studies related to gut microbiome during pregnancy or lactation are animal experiments. Therefore, there is a long journey before conclusion, the first step is to clarify the difference of gut microbiome between pregnant women with RA and healthy pregnant women. Then the specific bacteria associated with pregnancy with RA and its mechanism should be demonstrated. Finally, the method based on regulating gut microbiome to improve the disease activity of RA during pregnancy or lactation should be explored. The mechanisms of gut microbiome in RA during pregnancy and lactation may be (Figure 2): 1) The ability to produce citrullinization of peptides by enzymatic action. 2) Activation of antigenpresenting cells through an effect on TLRs or NLRs. 3) Antigenic mimicry 4) Control of the host immune system (triggering T cell differentiation). 5) Alterations in the permeability of intestinal mucosal. 6) Increase of T helper type 17-mediated mucosal inflammation.

In general, gut microbiome is a double-edged sword. Understanding the mechanism of gut microbiome in RA during pregnancy and lactation will provide potential and effective ideas for controlling the disease activity of RA. Mastering it will be beneficial to the treatment of RA during pregnancy or lactation.

## Conclusion

In summary, the conclusions of this paper are as follows: 1) Gut microbiome participates in the pathogenesis of RA and plays an important role in the development of RA. 2) There are significant differences in the diversity and abundance of gut microbiome during pregnancy and lactation compared with healthy women. 3) The disease activity of RA fluctuates abnormally during pregnancy or lactation, and the overall manifestation is aggravated, which affects the pregnancy plan. 4) Regulation of gut microbiome during pregnancy or lactation may be an effective treatment for RA. 5) Mechanisms of gut microbiome affecting the disease activity of RA during pregnancy or lactation may be related to the immune system, citrullinization of peptides and permeability of intestinal mucosal.

## Abbreviations

CIA, collagen-induced arthritis; DMARDs, disease-modifying antirheumatic drugs; FMT, fecal microbiota transplantation; GDM, gestational diabetes mellitus; MTX, methotrexate; NLRs, Nod-like receptors; RA, rheumatoid arthritis; SCFAs, short-chain fatty acids; SFB, segmented filamentous bacteria; SGA, small for gestational age; SSZ, sulfasalazine; Th1/Th2/Th17, T helper cell 1/2/17; TLRs, Toll-like receptors; TTP, time to pregnancy.
